# Whole-Genome Characterization and Strain Comparison of VT2f-Producing *Escherichia* *coli* Causing Hemolytic Uremic Syndrome

**DOI:** 10.3201/eid2212.160017

**Published:** 2016-12

**Authors:** Laura Grande, Valeria Michelacci, Roslen Bondì, Federica Gigliucci, Eelco Franz, Mahdi Askari Badouei, Sabine Schlager, Fabio Minelli, Rosangela Tozzoli, Alfredo Caprioli, Stefano Morabito

**Affiliations:** Istituto Superiore di Sanità, Rome, Italy (L. Grande, V. Michelacci, R. Bondi, F. Gigliucci, F. Minelli, R. Tozzoli, A. Caprioli, S. Morabito);; National Institute for Public Health and the Environment, Bilthoven, the Netherlands (E. Franz);; Faculty of Veterinary Medicine, Garmsar Branch, Islamic Azad University, Garmsar, Iran (M. Askari Badouei);; Austrian Agency for Health and Food Safety, Graz, Austria (S. Schlager)

**Keywords:** VT2f-producing, variant verotoxigenic Escherichia coli, virulence, evolution, enteric infections, bacteria, zoonoses, VTEC, Escherichia coli, whole-genome characterization, hemolytic uremic syndrome, HUS

## Abstract

Verotoxigenic *Escherichia coli* infections in humans cause disease ranging from uncomplicated intestinal illnesses to bloody diarrhea and systemic sequelae, such as hemolytic uremic syndrome (HUS). Previous research indicated that pigeons may be a reservoir for a population of verotoxigenic *E. coli* producing the VT2f variant. We used whole-genome sequencing to characterize a set of VT2f-producing *E. coli* strains from human patients with diarrhea or HUS and from healthy pigeons. We describe a phage conveying the *vtx2f* genes and provide evidence that the strains causing milder diarrheal disease may be transmitted to humans from pigeons. The strains causing HUS could derive from VT2f phage acquisition by *E. coli* strains with a virulence genes asset resembling that of typical HUS-associated verotoxigenic *E*. *coli*.

## Abstract 2

Strains from diarrheal illnesses could be transmitted from pigeons, but HUS-associated strains may derive from phage acquisition by isolates with larger virulence assets.

Verotoxigenic *Escherichia coli* (VTEC) infections in humans cause a wide spectrum of clinical manifestations ranging from uncomplicated forms of intestinal illnesses to bloody diarrhea and systemic sequelae, such as hemolytic uremic syndrome (HUS) (*1*). The most severe forms are caused by the damage inflicted by the verocytotoxins (VTs) to the target cells in the intestinal mucosa and the renal blood vessels (*1*). The genes encoding the verocytotoxins (*vtx*) are harbored by lambdoid bacteriophages, which can be transferred to multiple bacterial hosts, generating a great diversity in the bacterial types that produce such toxins (*2*).

The most well-known VTEC serogroup, O157, inhabits the gastrointestinal tract of ruminants, especially cattle. However, this and other VTEC serotypes have been isolated from the feces of several other animal species, including deer, pigs, horses, cats, dogs, and wild birds (*3*).

During a program aimed at the control of the pigeon population in Rome, Italy during 1998, G. Dell’Omo et al. observed that this animal species was a carrier of VTEC (*4*). In that study, VTEC of multiple serogroups were isolated from ≈10% of the animals tested. Of 16 VTEC, 15 carried the *eae* gene encoding the intimin and featured genetic determinants that produced a subtype of verocytotoxin type 2 not described before, later designated VT2f (*4*–*6*). The finding of such a high prevalence of VTEC in pigeons living in Rome led to further research into these bacteria in this and other bird species worldwide. Almost all these studies succeeded in isolating VTEC, with prevalence ranging 3% to >19% in different countries and bird species; most VTEC isolated from pigeon feces and cloacal swab samples harbored the genes encoding the VT2f subtype (*7*–*10*). These findings emphasize the existence of a strict association between VTEC carrying the *vtx2f* genes and pigeons, which represent a reservoir for such strains.

Data on human illness attributable to VT2f-producing *E. coli* has been scarce until recent reports from Germany and the Netherlands described the isolation of such strains from diarrheal stool specimens from humans (*11*,*12*). Furthermore, in the Netherlands, an HUS case was recently reported to be associated with the presence of a VT2f-producing O8:H19 strain (*13*). We aimed to characterize at the whole-genome level 3 *E. coli* strains that produced the VT2f isolated from HUS and to investigate their relationships with VT2f-producing *E. coli* isolated from human diarrheal cases and from the pigeon reservoir.

## Materials and Methods

### Bacterial Strains

We investigated 22 Vt2f -producing *E. coli* strains. Eight previously described strains were isolated from pigeons in Italy (*4*); eleven strains were isolated in the Netherlands from fecal specimens from humans with diarrhea during 2008–2012 and are part of the collections held at the National Institute for Public Health and the Environment in the Netherlands (RIVM) (*12*). Of the 3 VT2f-producing *E. coli* from HUS patients, 1 was isolated in Austria in 2013 and 2 in Italy during 2013–2014. A total of 23 unrelated VTEC non-O157 strains that produced VT1 and/or VT2 subtypes other than VT2f have been used for the comparison of the profiles of virulence genes with those of the VT2f-producing isolates (Table 1).

**Table 1:** Characteristics of non-O157 verotoxigenic *Escherichia* *coli* strains used in a comparative analysis of the virulence profile of VT2f-producing strains from humans and the animal reservoir*

Strain	Serogroup	Source†	Year of isolation	Virulence gene profile
ED017	O26	HUS	1989	*eae vtx1*
ED075	O26	Diarrheal feces	1990	*eae vtx1*
ED180	O26	HUS	1994	*eae vtx2*
ED195	O26	HUS	1994	*eae vtx1*
ED392	O26	Diarrheal feces	1998	*eae vtx1*
ED411	O26	HUS	1999	*eae vtx2*
ED423	O26	Diarrheal feces	1999	*eae vtx1*
ED654	O26	HUS	2007	*eae vtx2*
ED669	O26	HUS	2008	*eae vtx1*
ED676	O26	HUS	2008	*eae vtx2*
ED729	O26	Diarrheal feces	2010	*eae vtx1*
ED766	O26	HUS	2010	*eae vtx2*
ED657	O145	HUS	2007	*eae vtx2*
ED603	O121	HUS	2004	*eae vtx2*
ED073	O111	Diarrheal feces	1990	*eae vtx1*
ED082	O111	HUS	1990	*eae vtx1*
ED142	O111	HUS	1992	*eae vtx1 vtx2*
ED178	O111	HUS	1994	*eae vtx1 vtx2*
ED608	O111	HUS	2005	*eae vtx1 vtx2*
ED664	O111	HUS	2007	*eae vtx2*
ED672	O111	HUS	2008	*eae vtx1 vtx2*
ED287	O103	Bovine	1998	*eae vtx1*
ED728	O103	Bloody diarrheal feces	2010	*vtx1*
*All samples are from humans except strain ED287. HUS, hemolytic uremic syndrome. †HUS samples were isolated from feces.				

### Whole-Genome Sequencing of *E. coli* Strains

Sequencing of the strains isolated from fecal samples from humans with diarrhea and from pigeons was outsourced to the Central Veterinary Institute, Wageningen University (Lelystad, the Netherlands). Genome sequences were obtained by using a TruSeq protocol on an Illumina MiSeq PE300 platform (Illumina, San Diego, CA, USA). The genomes of the 3 VT2f-producing isolates from HUS patients were sequenced by using an Ion Torrent PGM (Thermo Fisher Scientific, Waltham, MA, USA) according to 400-bp protocols for library preparation through enzymatic shearing, Ion OneTouch2 emulsion PCR, enrichment, and Hi-Q sequencing kits (Thermo Fisher Scientific).

The whole-genome sequences (WGSs) of the 23 non-O157 VTEC strains are part of the European Molecular Biology Laboratory’s European Nucleotide Archive Study (http://www.ebi.ac.uk/ena; accession no. PRJEB11886). The raw reads have been subjected to quality check through FastQC and trimmed with FASTQ positional and quality trimming tool to remove the adaptors and to accept 20 as the lowest Phred value (*14*).

We subjected the sequences obtained with the Ion Torrent apparatus to de novo assembly by using the tool SPADES (*15*) and those from Illumina by using the A5 pipeline (*16*). The genomes have been assembled in several contigs ranging from 42 to 495 (mean 225), with N50 values (the length of the smallest contig among the set of the largest contigs that together cover at least 50% of the assembly) between 40,736 and 347,638 (mean 152,953). All the contigs were uploaded to the EMBL European Nucleotide Archive (accession no. PRJEB12203). We made annotations by using the Prokka tool (*17*). All the bioinformatics tools used are available on the Aries public Galaxy server (https://w3.iss.it/site/aries/).

### Virulence Gene Profile Analysis and Serotyping

The presence of *vtx2f* and *eae* genes has been assessed by PCR by using primers and conditions described elsewhere (*5*,*18*). The activity of VT2f has been evaluated by Vero cell assay (VCA) as previously described (*19*).

We performed detection of the virulence genes *cif, efa1, espABCFIJP, etpD, iha, iss, katP, lpfa, nleABC, tccP, tir, toxB, ehxA*, and *espP* and the serotype determination in silico on the WGSs. We used blastn (available on the Aries public Galaxy server at https://w3.iss.it/site/aries/) to search databases containing the reference sequences of all the known virulence and serotype-associated genes of pathogenic *E. coli* (*20*). To perform the principal component analysis of the virulence gene profiles, we used SAS/IML studio software version 3.4 (SAS Institute, Inc., Cary, NC, USA).

We investigated plasmid profiles by using PlasmidFinder (*21*; https://cge.cbs.dtu.dk//services/all.php). The intimin subtyping has been performed in silico through a BLAST search (*22*) of the *eae* gene sequences from the WGS against the National Center for Biotechnology Information nucleotide repository. The intimin types of the VT2f-producing strains isolated from pigeons have been published (*6*,*10*).

### *rpoB* Sequencing and Analysis

Amplification and sequencing of the *rpoB* gene were conducted to discriminate between *E. coli* and *E. albertii* species, as previously described (*23*). The amplicons were purified with the SureClean Plus kit (Bioline, London, UK) and sequenced using the BigDye Terminator v1.1 kit on a Genetic Analyzer 3130 (Thermo Fisher Scientific). The obtained sequences were trimmed and aligned to the reference sequences as indicated (*23*), using the Clustal Omega free software (http://www.ebi.ac.uk/Tools/msa/clustalo/).

### Typing

We determined *E. coli* phylogenetic groups by using the method of Clermont et al. (*24*). We carried out multilocus sequence typing (MLST) of the VT2f isolates in silico according to the scheme proposed by Wirth et al. (*25*). We analyzed the assembled sequences by using blastn to search the MLST database downloaded from the Internet site of the MLST.UCC Mark Achtman database (http://mlst.warwick.ac.uk/mlst/dbs/Ecoli/).

### Single-Nucleotide Polymorphism (SNP) Analysis

We analyzed SNPs by using the tool kSNP3 (*26*) available on the Galaxy project instance Aries (https://w3.iss.it/site/aries/). We set a kmer value of 23.

## Results

### Characterization of the VT2f-Producing *E. coli* Strains

#### Serotyping

Of 11 VTEC strains isolated from humans with diarrhea, 5 belonged to the O63:H6 serotype. The remaining 6 isolates contained the *fliC*_H6_ (3 strains), *fliC*_H7_ (1 strain), and *fliC*_H34_ (2 strains) genes (Table 2) and belonged to serogroups O96, O113, O132, O145, and O125. For 1 isolate, the O-antigen–associated genes could not be identified (Table 2) (*12*).

**Table 2:** Characteristics of VT2f-producing *Escherichia* *coli* investigated in a comparative analysis of the virulence profile of strains isolated from humans with mild and severe disease and from the animal reservoir*

Source and strain	Year isolated	Serotype	Phylotype	MLST	LEE	*adfO*	*efa1*	*cif*	*nleA*	*nleB*	*nleC*	*Hly*	*katP*	*espP*	Intimin type
Human diarrhea															
M856	2008	ONT:H6	B2	ST583	+	+	–	+	–	+	+	–	–	–	α-2
M858	2008	O125:H6	B2	ST583	+	+	–	+	–	+	–	–	–	–	α-2
M859	2009	O113:H6	B2	ST121	+	+	–	+	–	–	–	–	–	–	α-2
M884	2011	O96:H7	B2	ST28	+	+	–	+	+	+	–	–	–	–	β-2
M885	2011	O132:H34	B2	ST582	+	+	–	–	–	+	+	–	–	–	β-2
M900	2012	O145:H34	B2	ST722	+	+	–	–	–	+	–	–	–	–	ι
BCW5711	2012	O63:H6	B2	ST583	+	+	–	+	+	–	+	–	–	–	α-2
BCW5746	2012	O63:H6	B2	ST583	+	+	–	+	–	–	+	–	–	–	α-2
BCW5743	2012	O63:H6	B2	ST583	+	+	–	+	–	–	+	–	–	–	α-2
BCW5739	2012	O63:H6	B2	ST583	+	+	–	+	–	–	+	–	–	–	α-2
BCW5717	2012	O63:H6	B2	ST583	+	+	–	+	–	–	+	–	–	–	α-2
Pigeon															
ED360	1997	O45:H2	B1	ST20	+	+	–	+	+	+	+	–	–	–	β
ED361	1997	O75:H2	B1	ST20	+	+	–	+	+	+	+	–	–	–	β
ED363	1997	O4:H2	B1	UNK	+	+	–	+	+	+	+	–	–	–	β
ED366	1997	ONT:H2	B1	ST2685	+	+	–	+	+	+	+	–	–	–	β
ED369	1997	O45:H2	B1	ST20	+	+	–	+	+	+	+	–	–	–	β
ED377	1997	O4:H2	B1	UNK	+	+	–	+	+	+	+	–	–	–	β
ED430	2000	O45:H2	B1	ST20	+	+	–	+	+	+	+	–	–	–	β
ED444	2000	O128:H2	B1	ST20	+	+	–	+	+	+	+	–	–	–	β
HUS															
EF453	2013	O80:H2	B1	ST301	+	+	+	–	+	+	+	+	–	+	ξ
EF467	2013	O26:H11	B1	ST21	+	+	+	+	+	+	+	+	+	+	β
EF476	2014	O55:H9	B1	ST301	+	+	+	–	+	+	+	+	–	+	ξ

*Human samples were diarrheal or fecal samples from HUS cases and pigeon samples were feces from asymptomatic birds. LEE, locus of enterocyte effacement; MLST, multilocus sequence type; UNK, unknown; +, positive; –, negative.

Molecular serotyping of the 8 VT2f-producing strains isolated from pigeons showed that all the isolates had the *fliC_H2_* and the O4, O45, O75, and O128 serogroup-associated genes. The O-antigen genes could not be identified for the isolate ED 366 (Table 2). The HUS-associated VT2f-producing *E. coli* strains EF453 and EF476 belonged to serotypes O80:H2 and O55:H9, respectively, while strain EF467 was O26:H11.

#### Virulence Gene Profiles

The *E. coli* strains carrying the *vtx2f* and isolated from pigeons have been previously reported to produce an active VT2f (*6*). As expected, culture supernatants from VT2f-producing strains isolated from human diarrhea and HUS induced a cytopathic effect on Vero cells morphologically compatible with that caused by verocytoxins.

All the VT2f-producing strains included in the study were positive for the *eae* gene (Table 2) and displayed the presence of the entire locus of enterocyte effacement (LEE) (data not shown). Most of the *E. coli* VT2f-producing strains isolated from diarrheal cases harbored the α-2 intimin type (8/11), followed by the β-2 (2/11) and ι (1/11) types. The VT2f-strains isolated from pigeons had been previously described to have the β-intimin (*6*) in most cases and, more rarely, the α-2 intimin type (*10*). Of 3 HUS-associated VT2f-producing strains, 2 (EF453 and EF476) carried the ξ intimin type and 1 (EF467) had the β intimin (Table 2).

All the pigeon and HUS isolates possessed the complete set of non–LEE-encoded effectors assayed (*nleA, nleB* and *nleC*) (*27*), whereas the isolates from human diarrhea cases displayed an unequal presence of these genes (Table 2). The *efa1* gene, hallmark of the OI-122 pathogenicity island (*28*), was not identified in the isolates from pigeons or from human diarrheal specimens; neither were the genes *ehxA*, *espP* and *katP*, usually present on the large virulence plasmid of VTEC O157 and other VTEC associated with severe human disease (Table 2). However, the gene *adfO*, present on the OI-57 (*29*), was detected in all the strains investigated (Table 2).

The HUS strains EF453, EF467, and EF476 had the entire *efa1* gene. Strain EF467 also had the *ehxA*, *espP*, and *katP* genes; the EF453 and EF476 strains had the *ehxA* and *espP* genes only (Table 2). The analysis of the plasmid profiles substantiated the finding that the 3 HUS-associated strains carried the large virulence plasmid of VTEC, revealing the presence of a sequence 100% homologous to the replicon sequence of the pO26-CRL plasmid from a VTEC O26:H- (GenBank accession no. GQ259888.1), which harbors the genes *ehxA*, *espP*, and *katP*.

On the basis of plasmid profiles analysis, 7 of 11 *E. coli* VT2f-producing strains isolated from human diarrheal feces seemed to have the replicon sequence of the plasmid pSFO (GenBank accession no. AF401292) encoding the enterohemolysin and a cluster of *pap*-like genes called *sfp* in a sorbitol-fermenting *E. coli* O157 (*30*). However, the analysis of the WGSs failed to identify the *ehxA* and the *pap*-like sequences, suggesting that the entire pSFO plasmid was not present.

**Figure 1:**
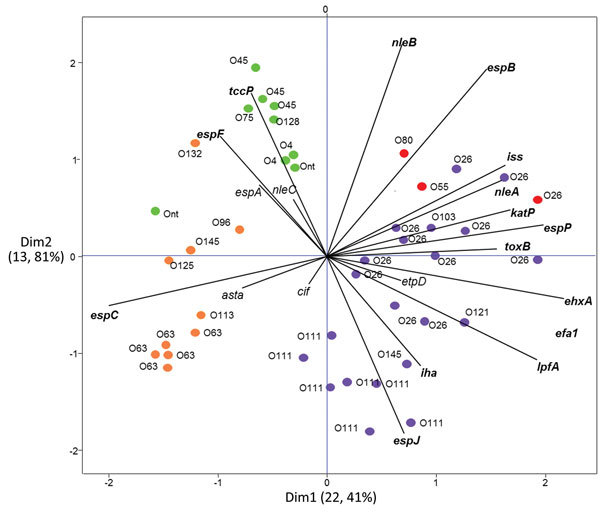
Principal component analysis of the virulence profiles of VT2f-producing verotoxigenic *Escherichia* *coli* (VTEC) strains isolated from fecal samples from human uncomplicated diarrheal case-patients (orange), human hemolytic uremic syndrome patients (red), and pigeon feces (green). Non-O157 VTEC that do not produce VT2f are indicated in purple. This analysis clusters isolates on the basis of similarities in virulence gene content. Isolates clustering together have similar virulence profiles. Lines represent individual virulence genes. The projected length of the lines on the horizontal and vertical axes indicates the strength of the contribution of a specific virulence marker to the separation of clusters. Dim1 and Dim2 represent the first 2 dimensions of the analysis and indicate the fraction of variation in the dataset that can be explained by the included variables (i.e., virulence genes).

Principal component analysis of the virulence genes profiles showed that the HUS isolates producing VT2f clustered with the set of non-O157 VTEC isolates used for comparison, rather than with the other VT2f-producing strains (Figure 1). Conversely, the VT2f-producing strains from diarrhea and from pigeons grouped together and apart from the HUS strains (Figure 1).

### Phylogenetic Analyses

#### rpoB Analysis

All the VT2f-producing isolates had an *E. coli-*related *rpoB* sequence (*23*). This finding verified that all the strains investigated were *E. coli*.

#### Typing

All VT2f-producing *E. coli* isolates from pigeons and the strains isolated from HUS belonged to the B1 phylogenetic group. All the strains isolated from human diarrheal feces were of phylotype B2 (Table 2).

By MLST, most of the pigeon strains investigated (5/8) belonged to sequence type (ST) 20; 1 was ST2685, and 2 were of unknown ST (Table 2), mainly because of the absence of a recognizable *adk* gene sequence. The 5 O63:H6, the 1 O125:H6, and the 1 ONT:H6 VTEC strains from diarrheal fecal specimens belonged to ST583; of the remaining 4 strains, 1 each was of sequence types ST28, ST121, ST582, and ST722 (Table 2).

Of 3 HUS-associated VT2f-producing *E. coli*, 2 (EF453 and EF476) belonged to ST301; strain EF467 was of ST21 (Table 2). All of the STs belonged to different clonal complexes or to any clonal complex, indicating that they were not related each other (data not shown).

### SNP Analysis

**Figure 2:**
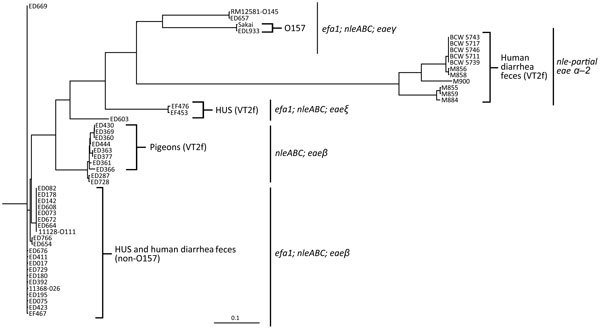
Parsimony tree obtained from the core-genome single-nucleotide polymorphism analysis of VT2f-producing verotoxigenic *Escherichia* *coli* (VTEC) strains from this study compared with VTEC belonging to the most relevant serogroups with publicly available sequences. In detail: 2 VTEC O157:H7 strains, EDL933 and Sakai (GenBank accession nos. NZ_CP008957.1 and NC_002695.1); 1 O26:H6, strain 11368 (accession no. NC_013361.1); 1 O145:H28, strain RM12581 (accession no. NZ_CP007136.1); and 1 O111:H-, strain 11128 (accession no. NC_013364.1). Scale bar indicates nucleotide substitutions per site.

A parsimony tree representing the core-genome SNPs analysis (Figure 2) shows that VT2f-producing strains from pigeons, human diarrheal feces, and HUS cases cluster apart from each other and from other VTEC strains used for comparison. The HUS-associated EF467 strain clusters together with the group of VTEC non-O157 from human disease, in agreement with the principal component analysis (Figures 1, 2).

### Identification of a Bacteriophage Containing the *vtx2f* Genes

The contigs containing the *vtx2f* genes in the different strains ranged 2,500–68,480 bp in size. Upon annotation, they showed the presence of phage-associated genes in the proximity of *vtx2f*, including those encoding the antitermination protein Q, the lysis protein S, a phage terminase, an integrase, and tail-assembly proteins.

We used WGS of HUS strain EF467 to assemble a partial VT2f phage sequence of 38,594 bp (Technical Appendix). Analysis of the construct highlighted the absence of most of the genes normally involved in the regulation of the switch between the lysogenic state and the lytic cycle of lambdoid phages, such as *cro*, *cI*, *cII*, *cIII*, and N. We confirmed these findings by mapping the raw reads of the WGS of the strain EF467 against the VT2 reference bacteriophage BP933W (GenBank accession no. AF125520) (not shown).

We confirmed the genomic structure of VT2f phage obtained in silico by 4 PCRs performed on the total DNA extracted from strain EF467 by using primer pairs designed on the construct’s map and by using a restriction fragment length polymorphism analysis on the obtained PCR fragments (Table 3; Technical Appendix). The partial VT2f phage sequence was deposited into the EMBL database (accession no. LN997803).

**Table 3:** PCR and restriction fragment length polymorphism analysis conditions used to verify VT2f phage structure in a comparative analysis of the virulence profile of human and zoonotic VT2f-producing *Escherichia* *coli* strains*

Analysis	Primer name	Sequence, 5′→3′	Position	Thermal profile	Amplicon size, bp	Restriction enzyme (obtained fragments, bp + bp)
PCR1	φ-*vtx2f*_1FW	caccatatcccagcaactgc	1,985–2,005	95°C for 2 min, 30× (94°C for 30 s, 53°C for 30 s, 70°C for 9 min); 72°C for 10 min	6,331	*Pvu*I (1,773 + 4,558)
	φ -*vtx2f*_1RV	gttggcggttccgactacaa	8,315–8,296			
PCR2	φ -*vtx2f*_2FW	gcgcatcaccacttcatctt	8,337–8,357	95°C for 2 min, 30× (94°C for 30 s, 53°C for 30 s, 70°C for 9 min), 72°C for 10 min	8,166	*Hind*III (1,855 + 6,311)
	128–1	agattgggcgtcattcactggttg	16,502–16,479			
PCR3	φ -*vtx2f*_3FW	ggagtggatattgccgacct	16,808–16,827	95°C for 2 min, 30× (94°C for 30 s, 53°C for 30s, 70°C for 9 min), 72°C for 10 min	3,927	*BgI*II (1,310 + 2,617)
	φ -*vtx2f*_3RV	gtcttcctgctgaggcgatc	20,734–20,715			
PCR4	φ -*vtx2f*_4FW	taatcgcggccgtactcaag	22,172–22,191	95°C for 2 min, 30× (94°C for 30 s, 53°C for 30 s, 70°C for 9 min), 72°C for 10 min	8,808	*Nco*I (5,029 + 3,779)
	φ -*vtx2f*_4RV	tgttcagctccaccttacgg	30,979–30,960			

*Analysis for PCR2, primer 128–1 from (*5*); all other data were compiled for this study. All the long PCR described were performed with the GoTaq Long PCR Master Mix (Promega, Madison, WI, USA) according to manufacturer’s instructions. Primer positions refer to the phage sequence deposited into the EMBL database (accession no. LN997803).

The portion of the VT2f phage spanning the *xerC* gene and the tRNA-Gly and tRNA-Thr loci (Technical Appendix) was found in the draft genomes of all the VT2f-producing strains. The region downstream to the tRNA loci was also detected, but with different degrees of variation among the WGSs; for example, the presence of an additional DNA stretch of ≈24 kb in the strain BCW5746 (Technical Appendix).

**Figure 3:**
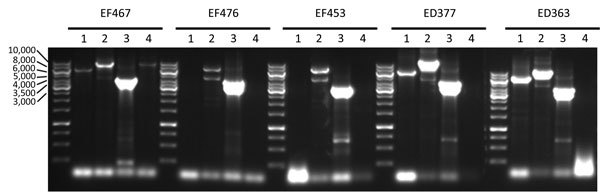
Long PCR analysis of the VT2f phage of verotoxigenic *Escherichia* *coli* (VTEC) strains isolated from fecal samples from humans with hemolytic uremic syndrome (EF467, EF476, EF453) and from pigeon feces (ED363, ED377). Numbers at left indicate bps; lane numbers indicate PCR 1 to PCR 4. The expected size of the amplicons were 6,331 bp (PCR 1), 8,166 bp (PCR 2), 3,927 bp (PCR 3), and 8,808 bp (PCR 4).

The presence of a similar phage structure was confirmed by using a long PCR approach in the other 2 VT2f-producing *E. coli* from HUS cases and in 2 strains from pigeons (strains ED377 and ED363). All the strains showed the expected amplicons with PCR 2 and 3 together with the expected product of PCR 1 in 2 pigeon isolates; all the isolates tested did not yield any amplicon with PCR 4 (Figure 3). A comparative analysis of the *xerC* sequences from all the VT2f phage constructs returned a high degree of variation in its sequence, explaining the observed absence of the PCR4-specific amplicon (data not shown). Finally, a BLAST search by using this VT2f phage construct from strain EF467 returned only partial similarity with phages identified in different *Enterobacteriaceae* but did not retrieve similar structures.

## Discussion

VTEC producing the VT2f subtype have long been considered a minor public health problem because of their rare association with human infections (*31*–*34*). Recently, however, an increasing number of reports of human diseases caused by infection with these *E. coli* strains have populated the literature (*11*,*12*,*32*,*35*). The diversity of *vtx2f* gene sequences compared with other *vtx2* subtype genes may have played a role in underestimating the global burden of such infections. PCR primers mostly used for the detection of *vtx2* genes in clinical specimens and the vehicles of infection have been proven to be unable to amplify the *vtx2f* gene (*36*). In addition, the recent description of another *eae*-positive *Escherichia* species often isolated from birds, and sometimes carrying *vtx2f* genes, *E. albertii*, added a further element of confusion. *E. albertii* has been associated both with gastroenteritis in humans and with healthy and diseased birds (*37*,*38*), but this species is difficult to distinguish from *E. coli* when using the usual biochemical or molecular assays.

Most human infections with VTEC producing VT2f have been reported as uncomplicated diarrheal cases (*11*,*12*), which may also have accounted for the underestimation of these infections. Because such cases are not actively surveyed in many countries, these infections may have been overlooked. The recent description of an HUS case associated with a VT2f-producing *E. coli* (*13*) changed the perspective on VT2f-producing *E. coli* and the associated disease, making it necessary to update the current paradigm of HUS-associated VTEC.

We provide evidence that the VT2f-producing *E. coli* isolated from HUS cases display the complete set of virulence genes described in the typical HUS-associated VTEC (Table 2; Figure 1) (*28*). All VT2f strains from HUS that we examined were positive for pathogenicity island OI-122 (*28*) and the large virulence plasmid first described in VTEC O157 (Table 2) (*39*); the strains from pigeons or from humans with uncomplicated diarrhea did not have these virulence-associated mobile genetic elements (Table 2) (*10*–*12*,*32*,*40*).

Our study also showed that the LEE was complete in all the genomes investigated, but a complete set of *nleABC* genes was found only in strains from pigeons and from humans with HUS (Table 2), indicating that the VT2f-producing isolates investigated belonged to 3 distinct main virulotypes or subpopulations (Table 2). The intimin subtyping supported this observation. Of 11 diarrheal isolates, 8 had the α-2 gene; all the pigeon isolates had a β intimin coding gene. Finally, 2 of the 3 strains from HUS showed the presence of a gene encoding the ξ intimin (Table 2). Furthermore, the analysis of core genome SNPs confirmed the existence of different subpopulations of VT2f-producing *E. coli* (Figure 2). The analysis of the virulence genes suggests that different populations of VT2f-producing *E. coli* exist and have different potential to cause human disease on the basis of the virulotype to which they belong.

VT2f-producing *E. coli* strains isolated from uncomplicated human cases of diarrhea have been reported in the literature as being ST20 (*11*), which is the same sequence type we identified in most pigeon isolates; this ST was also described in VT2f-producing *E. coli* isolated from pigeons in Japan (*40*). The same study also described an animal isolate of ST722, which was found in 1 strain isolated from human diarrheal feces in our study (Table 2). Similarly, the serotypes in some cases appeared to overlap isolates from pigeons and human cases of diarrhea, such as the serotype O128:H2 that we found in 1 pigeon isolate that was also reported in isolates from human cases of diarrhea in Germany (*11*).

Altogether, these observations indicate that the VT2f-producing *E. coli* causing diarrhea in humans could be a subpopulation of those inhabiting the pigeon reservoir. Alternately, information on the serotypes, ST, and principal component analysis of virulence genes profiles supports the hypothesis that the HUS VT2f-producing strains are more similar to the non-O157 VTEC often isolated from samples from humans with severe disease (Figure 1) than to the other VT2f-producing *E. coli* from humans with diarrhea or from asymptomatic pigeons. This hypothesis suggests that the HUS VT2f-producing strains represent a distinct population of VTEC; whether they are part of the pigeon intestinal flora or arise from an acquisition of the *vtx2*-phage is difficult to ascertain. 

The phylogeny of VTEC of different serogroups, investigated by core SNP analysis, showed that the different VT2f-producing *E. coli* cluster into different subpopulations that include strain EF467 grouping together with non-O157 VTEC strains from humans with disease (Figure 2). However, the results from SNP analysis for VTEC of multiple serogroups should be carefully evaluated; the population structure of VTEC belonging to serogroups other than O157 and O26 has not been completely investigated yet.

At the first characterization of the *vtx2f* genes, it was proposed that they were, similar to other VT-coding genes, located on bacteriophages (*5*). Our study confirms this hypothesis and shows that such a phage apparently does not have similar counterparts in the VT-phage genomes reported in the National Center for Biotechnology Information nucleotide repository (http://www.ncbi.nlm.nih.gov/). In addition, we observed that VT2f phage was very similar in all the VT2f-producing *E. coli* investigated (Figure 3; Technical Appendix), suggesting that the *vtx2f* genes are present in phages sharing a common ancestor that is different from other phages with the other *vtx1*/*vtx2* subtypes.

In conclusion, we provide evidence that human infections with VT2f-producing *E. coli* are zoonotic diseases transmitted from pigeons. Such an animal reservoir may either directly disseminate VTEC strains causing diarrhea or indirectly release VT2f phages in the environment, which can in turn lysogenize *E. coli* strains that contain accessory virulence determinants and confer them the ability to cause HUS. The isolation of VT2f-producing *E. coli* with a virulence gene profile related to the other HUS-associated VTEC suggests that the severity of the symptoms induced by infection may depend more on the ability to achieve a proficient colonization of the host gut mucosa rather than on the subtype of the produced toxin.

Technical Appendix Maps of partial VT2f phages constructed by using the whole-genome sequences of verotoxigenic *Escherichia* *coli* strains.
