# Supplementary material for: Whole-Genome Characterization and Strain Comparison of VT2f-Producing Escherichia coli Causing Hemolytic Uremic Syndrome
Source: Emerg Infect Dis. 2016 Dec;22(12):2078–86. doi: 10.3201/eid2212.160017 (PMC5189129; doi:10.3201/eid2212.160017)
Supplement: Technical Appendix — Maps of partial VT2f phages constructed by using the whole-genome sequences of verotoxigenic Escherichia coli strains. [file 16-0017-Techapp-s1.pdf]

Article DOI: http://dx.doi.org/10.3201/eid2212.160017

Whole Genome Characterization and Strain Comparison of VT2f-Producing *Escherichia coli* Causing Hemolytic Uremic Syndrome

Technical Appendix

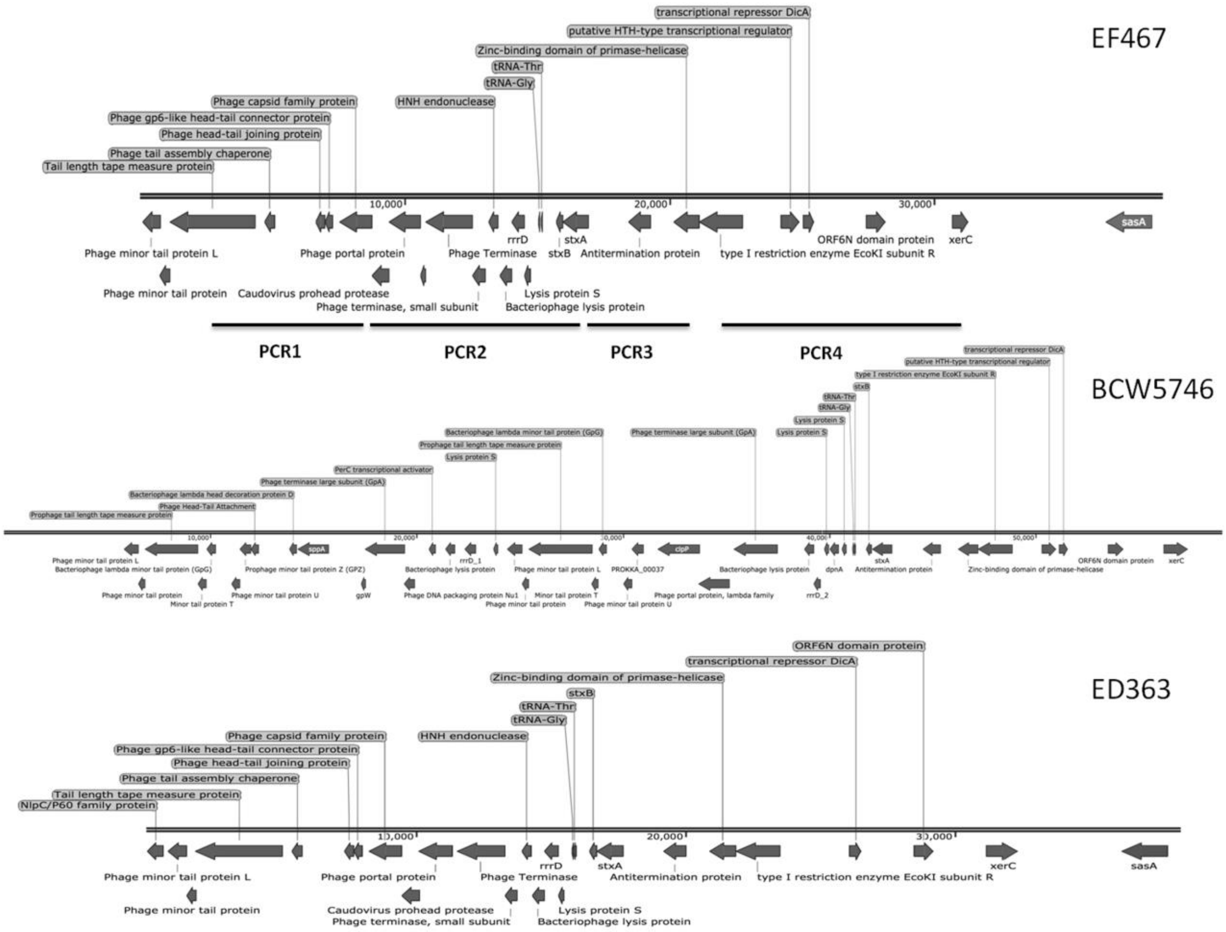

Technical Appendix Figure. Maps of the partial VT2f phages constructed by using the whole-genome sequences of the verotoxigenic *Escherichia coli* strains EF467, isolated from fecal specimens from a patient with hemolytic uremic syndrome; BCW5746, isolated from fecal specimens from a patient with diarrhea; and ED363, isolated from feces specimens from pigeons. The long PCRs used to verify the phages' structures are indicated as black bars. orf, open reading frame.
